# Circular RNA in pancreatic cancer: a novel avenue for the roles of diagnosis and treatment

**DOI:** 10.7150/thno.56174

**Published:** 2021-01-01

**Authors:** Zeyin Rong, Jin Xu, Si Shi, Zhen Tan, Qingcai Meng, Jie Hua, Jiang Liu, Bo Zhang, Wei Wang, Xianjun Yu, Chen Liang

**Affiliations:** 1 Department of Pancreatic Surgery, Fudan University Shanghai Cancer Center, Shanghai, 200032, China.; 2 Department of Oncology, Shanghai Medical College, Fudan University, Shanghai, 200032, China.; 3 Pancreatic Cancer Institute, Fudan University, Shanghai, 200032, China.; 4 Shanghai Pancreatic Cancer Institute, Shanghai, 200032, China.

**Keywords:** Circular RNA, pancreatic cancer, sponge function, biomarker, treatment

## Abstract

Pancreatic cancer (PC), an important cause of cancer-related deaths worldwide, is one of the most malignant cancers characterized by a dismal prognosis. Circular RNAs (circRNAs), a class of endogenous ncRNAs with unique covalently closed loops, have attracted great attention in regard to various diseases, especially cancers. Compelling studies have suggested that circRNAs are aberrantly expressed in different cancer tissues and cell types, including PC. More specifically, circRNAs can modify the proliferation, progression, tumorigenesis and chemosensitivity of PC, and some circRNAs could serve as biomarkers for diagnosis and prognosis. Herein, we summarize what is currently known to be related to the biogenesis, functions and potential roles of human circRNAs in PC and their application prospects for PC clinical treatments.

## Introduction

As one of the most malignant tumors with a dismal prognosis, pancreatic cancer (PC), ranks fourth in cancer mortality in the United States, and accounts for 8% of all estimated cancer-related deaths 1, 2, 3. Even worse, it is emerging as the second primary cause of cancer-related deaths by 2030 1, 2, 3. PC is characterized by rapid progression, high metastasis and recurrence, and fast development of drug resistance. Despite numerous efforts to improve the efficacy of surgery and chemoradiotherapy over the last several years, there are still few reliable biomarkers or notably better therapeutic strategies for daily clinical practice in PC 4. Consequently, it is absolutely essential to determine the potential mechanisms of cancer initiation and development for the diagnosis and treatment of pancreatic cancer.

CircRNAs are a group of endogenous noncoding RNAs that were originally misinterpreted as the products of splicing errors 5. Currently, it has been clarified that circRNAs are generated from introns or exons through back-splicing and possess a covalently closed circular loop without 5' end caps and 3' polyadenylated tails 6. Most of them are widespread, abundant, conserved, and stable and have tissue/developmental-stage-specific characteristics in eukaryotes 7, 8. CircRNAs are more resistant to exonuclease-mediated degradation and regular mechanisms of linear RNA decay than linear RNAs because of their unique single-stranded closed circle loop, and are not sensitive to regular mechanisms of linear RNA decay because of their unique single-stranded closed circular loop, which contributes to their abundance in tissues, serum, and urine and makes them promising biomarkers for aging and human cancers 9, 10. Currently, circRNAs have garnered much scientific attention because of their aberrant expression and pivotal impacts on the regulation and pathogenesis of different diseases, particularly human tumors 11. Increasing evidence indicates that circRNAs have considerable functional potential to alter proliferation, invasion, apoptosis, metastasis, angiogenesis, and the response to chemotherapy, indicating that circRNAs may function as novel potential therapeutic targets for the treatment of various tumors 12, 13, 14, 15, 16, 17, including pancreatic cancer 18, 19, 20, 21. For instance, a landmark discovery confirmed the exonic circRNA ciRS-7 (CDR1as), which contains over seventy binding sites for miR-7 9, as the first functional circRNA. CiRS-7 plays an oncogenic role by binding with miR-7 and elevating the expression of its downstream oncogenes in pancreatic cancer 18.

In this review, we summarize the current understanding of the biogenesis and functions of circRNAs and their roles in pancreatic cancer and discuss the application prospects of circRNAs for pancreatic cancer clinical treatments.

## Classification and biogenesis of circRNAs

**Figure 1:**
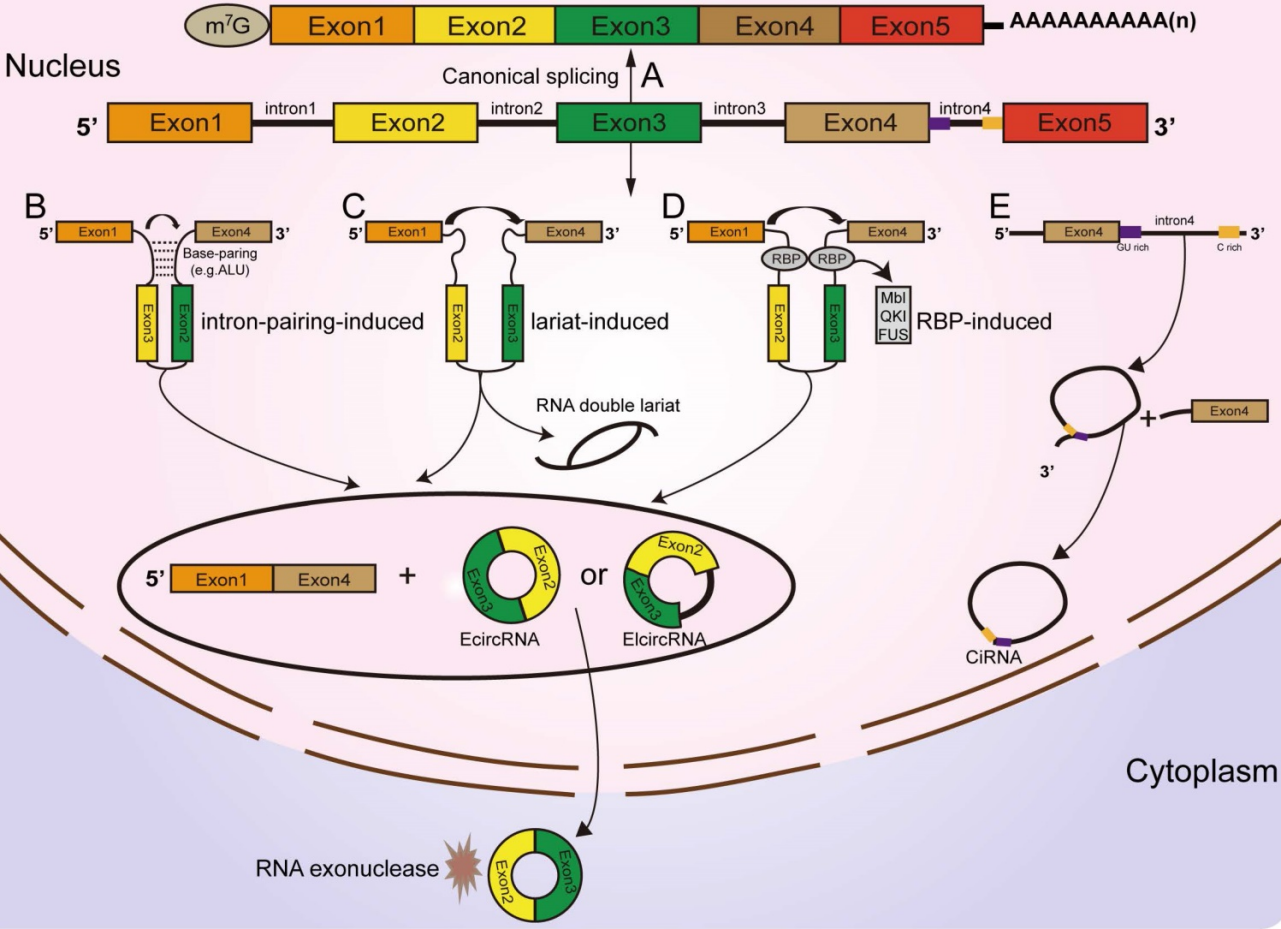
** Classification and biogenesis of circRNAs (A)** Canonical splicing related to linear RNA generation. **(B)** Intron-pairing circularization: the 3' splicing receptor site in the exon combines with the 5' splicing donor site, with the assistance of base pairing between intronic repeats, resulting in spatial proximity of the donor and acceptor splice exons. **(C)** Lariat-induced circularization: the 5' splice site attacks the 3' splice site, and skipped exons 2 and 3 are connected by producing a lariat. **(D)** RBP-induced circularization: RBPs bind to introns near splice sites, which can bridge flanking introns together, facilitating the production of circRNAs. **(E)** CiRNA formation: ciRNAs are produced from lariat introns. The GU-rich motif close to the 5' splice site (purple box) and the C-rich motif close to the branchpoint site (yellow box) prevent the intron from debranching and form a stable circRNA.

According to their origin and genomic organization, circRNAs are classified into 3 groups: exonic circRNAs (EcircRNAs, produced from exons), exon-intron circRNAs (EIcircRNAs, produced from exons and introns), and intronic circRNAs (ciRNAs, produced from introns) 22, 23, 24. Unlike the mechanisms of linear RNA generation **(Figure 1A)**, the specific formation mechanism of circRNAs is not yet fully clarified. As the most abundant circRNAs, EcircRNAs are derived from a specific splicing mechanism known as exon back-splicing. An upstream 5' splice donor attacks the downstream 3' splice acceptor, leading to the formation of a covalently closed circRNA 25. When the introns located in the 5' donor and the 3' acceptor on the precursor mRNA (premRNA)are retained 25, the resultant circRNAs are called EIcircRNAs and are composed of both exons and introns. Existing studies have proposed three biogenesis models of the synthesis of circRNAs: intron-pairing circularization, lariat- induced circularization, and RNA-binding protein (RBP)-induced circularization 26. First, for intron-pairing circularization, the complementary sequences from the introns in premRNAs generally facilitate the circularization of EcircRNAs 25. By means of base pairing between intronic repeats, the 3' splice acceptor site on the exon combines with the 5' splice donor site, leading to spatial proximity of the downstream and upstream splice exons 27, which facilitates exon circularization to form EcircRNAs** (Figure 1B)**. A previous study demonstrated that very short introns with lengths of 30-40 nucleotides comprising repeat complementary interdependent sequences located in upstream and downstream introns (such as Alu repeats) could be used to interfere with exon circularization after extensive mutagenesis of expression plasmids in human cells 25, 28. In lariat-driven circularization, the splice sites of skipped exons are connected to generate a lariat during the transcription of premRNA 25, 29, 30, 31. Then, the intronic sequences are removed from the lariats, and EcircRNAs are formed** (Figure 1C)**. In addition, RBPs are considered critical regulators of EcircRNA biogenesis 32, 33. They can specifically bind to the introns near splice sites and bring flanking introns closer together to facilitate the production of circRNAs **(Figure 1D)** 34. For example, the Drosophila Muscleblind (Mbl)-binding protein, derived from its second exon, can recognize and bind to particular motifs that are located in flanking introns on its own premRNA to increase circMbl production 35, 36. The flanking introns of circMbl contain highly conserved Mbl-binding elements 37, which can be recognized and precisely bound by the Mbl protein, thereby influencing the biogenesis of circMbl 35, 36. Quaking (QKI), an alternative splicing factor 38, can induce the production of hundreds of circRNAs by binding to recognition elements within introns and forming dimers, which can promote the efficiency of back-splicing during human epithelial-mesenchymal transition (EMT) 33. A study from Errichelli L et al. illustrated that the RBP fused in sarcoma (FUS) participated in circRNA biosynthetic processes by associating with the introns near splice junctions 39, 40. Under some circumstances, the end of the 2'-OH group of the intron and the 5' splice site form a branchpoint 2'-5' concatenation, after releasing the 3' exon 25.The ciRNAs are formed. Their structure depends on conserved elements, including seven-nucleotide GU abundant motifs near the 5' splice site and eleven-nucleotide C abundant motifs near the branchpoint site 41, 42, 43. These motifs can protect ciRNAs from debranching and degradation** (Figure 1E)** 22, 42.

## Online databases related to circRNAs

To advance the research of the multiple applications of circRNAs, many online databases related to circRNAs have been established, such as CircRNADb 44, TransCirc 45, CircBase 46, Circ2Traits 47, CIRCpedia v2 48, CircInteractome 49, CircNet 50, MiOncoCirc 51, TSCD (tissue-specific circRNA) 52, cancer-specific circRNA (CSCD) 53, exoRBase 54, and circR2Disease 55 **(Table 1)**. Combined with the advancement of biotechnology 42, these circRNA-related databases could help discover meaningful circRNAs, forecast the interactions between circRNAs and target molecules and translation potency, and investigate their functions in the processes of physiological and pathological development in different diseases.

## Functions of CircRNAs

### CircRNAs act as sponges of miRNAs

**Figure 2:**
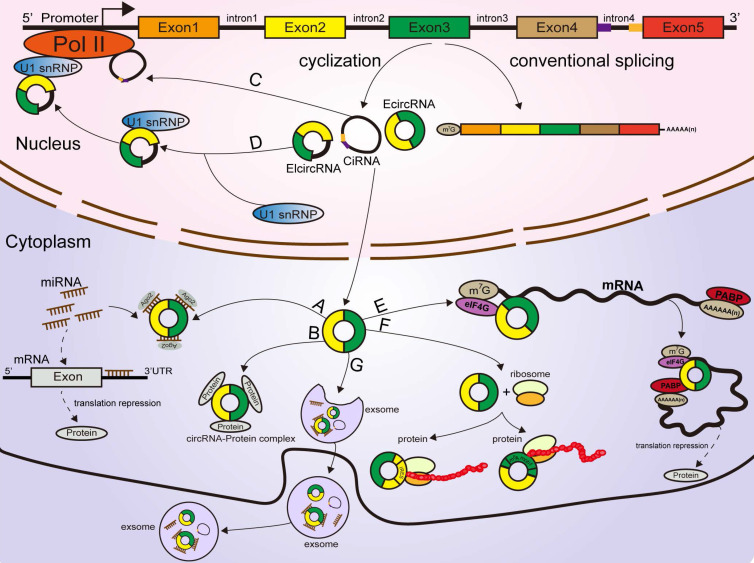
** Functions of circRNAs. (A)** CircRNAs function as miRNA sponges to influence the expression of downstream gene. **(B, C, D)** CircRNAs regulate parental gene expression at the transcriptional and translational levels. **(E)** CircRNAs bind with proteins to establish circRNA-protein complexes and alter the functions of some proteins. **(F)** CircRNAs encode proteins based on IRES-driven and m^6^A-driven models. **(G)** Some circRNAs carried by exosomes are derived from cancer cells.

Mechanistically, most identified circRNAs are mainly localized in the cytoplasm of the cell 56, indicative of their roles in posttranscriptional regulation 57. MiRNAs are a group of ubiquitous, conserved small noncoding RNAs with lengths of 19-25 nucleotides that can affect the expression of genes and a broad range of biogenesis functions in tumors 58. The ceRNA hypothesis indicates that circRNAs harbor MREs that bind miRNAs to reversely regulate the activity of the miRNAs 59, thus attenuating the inhibitory effect on their target molecules. Mounting evidence has confirmed that some circRNAs can repress miRNA function and modulate target gene expression to play a tumor suppressor or oncogenic role by acting as miRNA sponges in different cancers 15, 60, 61, 62. Furthermore, some circRNAs can also target multiple miRNAs and carry out opposite effects in different diseases. For instance, circ-ITCH suppresses the proliferation and progression of bladder cancer by absorbing miR-17 and miR-224 63. Moreover, circ-ITCH can also target miR-7 to elevate the level of EGFR and promote the migration and invasion of osteosarcoma 64. Recently, circBFAR was found to function for miR-34b-5p to promote the proliferation and metastasis of pancreatic ductal adenocarcinoma (PDAC) 65. In contrast, circNFIB1 directly sponge miR-486-5p to inhibit lymphangiogenesis and lymphatic metastasis of pancreatic cancer 66. Overall, acting as miRNA sponges may be a general function of circRNAs in tumors 67 **(Figure 2A)**.

### CircRNAs bind with proteins

Several circRNAs were revealed to bind with proteins to promote their functions. A ciRNA (circAGO2), produced from the premRNA of AGO2, physically interacts with the HuR protein and facilitates its transfer from the nucleus into the cytoplasm, leading to a decrease in AGO2 binding and suppression of the function of AGO2-miRNA complexes 68. Circ-Amotl1, derived from the Amotl1 gene, is predominantly expressed in the nucleus, where it colocalizes and interacts with the c-Myc protein. Upregulation of circ-Amotl1 in the nucleus facilitated translocation of the c-Myc protein into the nucleus and increased its binding affinity for some promoters 69, but did not alter the level of total c-Myc in breast cancer cells 69. CircECE1 binds to c-Myc and inhibits the degradation process mediated by SPOP, thereby reducing the efficiency of c-Myc modification and degradation by ubiquitination and promoting the stabilization of the c-Myc protein in osteosarcoma 70. Typically, circFOXK2 interacts with YBX1 and hnRNPK to increase the expression of the oncogenic proteins NUF2 and PDXK in PDAC 71.

Conversely, some circRNAs were confirmed to bind with proteins to suppress their functions 67. Recently, a study revealed that circSTAG1 binds with the well-known m6A demethylase ALKBH5, resulting in elevated m6A methylation of the mRNA of FAAH and its degradation in astrocytes 72. Circ-Foxo3, P21 and CDK2 form the ternary complex circ-Foxo3-P21-CDK2, which can suppress the progression of the cell cycle by inhibiting CDK2 function 73, 74, 75. All these findings suggest that circRNAs can act as decoys or scaffolds to affect the expression or function of some proteins 76 **(Figure 2B)**.

### CircRNAs affect parental gene expression

Emerging reports have shown that some circRNAs are retained in the cell nucleus to establish a large quantity of posttranscriptional regulatory factors that affect their parental gene expression 9. Zhang et al discovered that ci-ANKRD52 specifically interacts with the elongation RNA Pol II complex and directly promotes ANKRD52 transcription **(Figure 2C)**. Moreover, ci-ANKRD52 silencing reduced the transcription rate of ANKRD52 22. Another novel study discovered that circ-EIF3J and circ-PAIP2, identified as EIcircRNAs, can combine with RNA Pol II and facilitate their parental gene transcription in interaction with U1 snRNP in the nucleus of 293T and HeLa cells 77 **(Figure 2D)**. These findings suggest that circRNAs affect parental gene expression at the transcriptional level. Moreover, circRNAs can regulate gene expression at the translational level. For example, a complex is formed by the direct interaction of circYap (generated from the Yap gene), Yap mRNA, eIF4G and PABP, in which eIF4G and PABP are translation initiation-associated proteins. Overexpression of circYap in this complex inhibited the interaction of PABP with eIF4G and consequently suppressed the translation initiation of Yap mRNA **(Figure 2E)** 78. CircPABPN1 (hsa_circ_0031288), produced from the PABPN1 gene, suppressed HuR binding to PABPN1 mRNA and subsequently decreased the translation of PABPN1 79. All these discoveries indicated that circRNAs affect their parental gene expression at both the transcriptional and translational levels.

### CircRNAs encode proteins

Similar to most noncoding RNAs, circRNAs were originally considered untranslatable due to the lack of distinct ORFs. Nonetheless, emerging evidence corroborated that these so-called “noncoding RNAs” are translatable and can directly encode functional proteins **(Figure 2F)** 80, 81, 82, 83, 84, 85. Some EcircRNAs containing an IRES in the ORF have the ability to encode functional proteins or peptides in the cytoplasm 25, 44, 49, 86. For example, circFNDC3B translated a 218-amino acid novel protein (circFNDC3B-218aa) driven by an IRES 87. The junction-spanning ORF of circ-FBXW7 driven by an IRES encodes a novel 21-kDa protein, termed FBXW7-185aa, in glioblastoma 84, 88. Circβ-catenin has a putative IRES sequence and encodes a novel 370 aa β-catenin isoform that can stabilize β-catenin by inhibiting β-catenin phosphorylation and degradation induced by GSK3β in hepatocellular carcinoma 89, 90. Yang and coworkers revealed that in addition to IRES-mediated translation, a circRNA containing consensus N6-methyladenosine (m6A) motifs is translated 81. Moreover, this m6A-driven translation from the circRNA was decreased by FTO (m6A demethylase) and enhanced by METTL3/14 (adenosine methyltransferase) 81, 91. To date, circRNAs have been confirmed to be directly translated into proteins. However, more information is required to clarify the mechanism of circRNA translation to better understand other aspects of gene regulation.

## CircRNAs and pancreatic cancer

**Figure 3:**
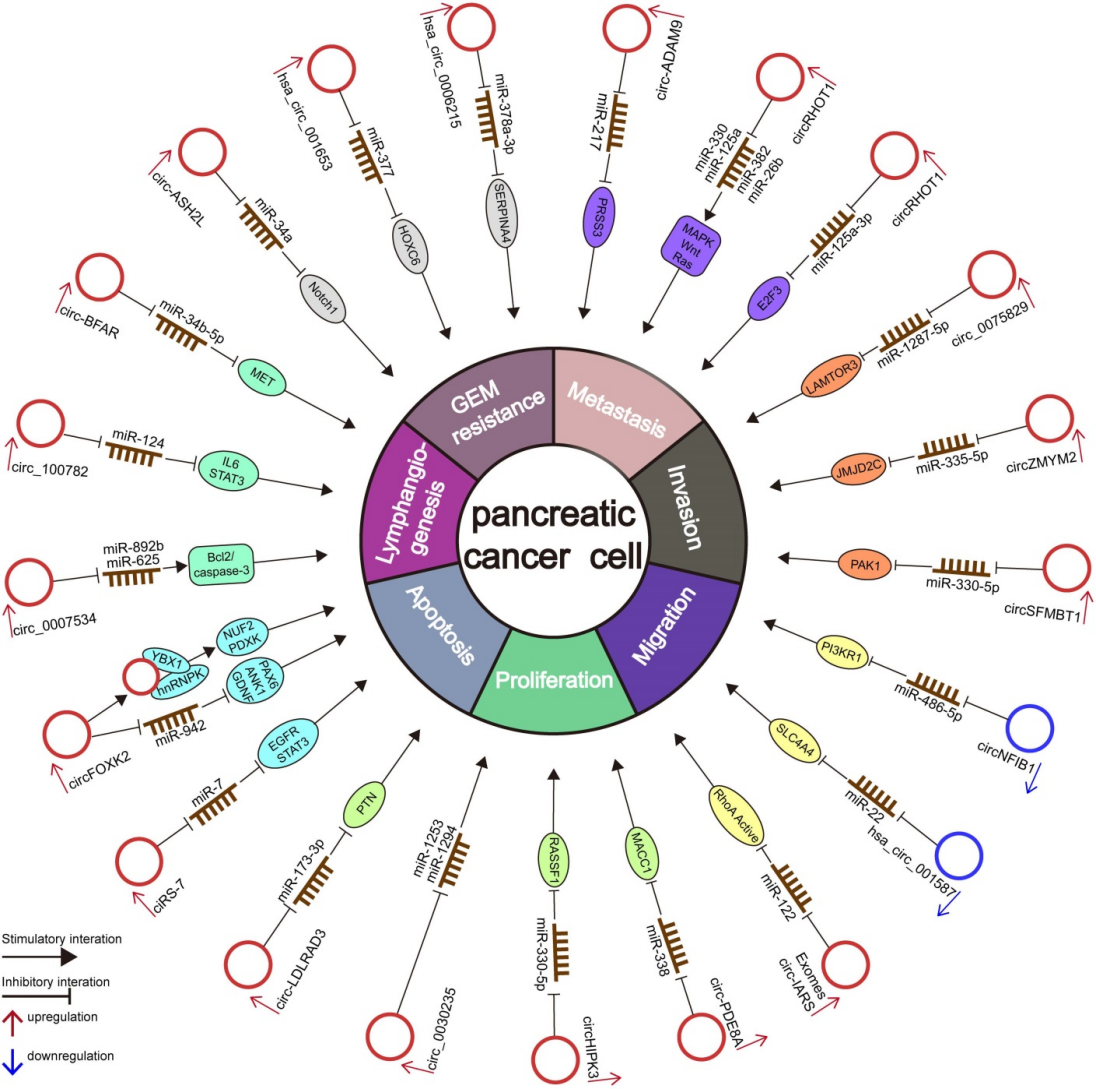
The diagram illustrates the mechanism underlying circRNAs in the regulation of cellular properties and miRNA-related gene regulation networks in PC.

Numerous circRNAs are abnormally expressed in several cancer types and exhibit a high degree of tissue- or disease-specificity differences 92, indicating that circRNAs may be used for diagnostic and therapeutic applications 51, 93. Although considerable studies have confirmed that circRNAs play essential roles in the occurrence and progression of PC, the study of the potential correlation and potential mechanism between circRNAs and PC is still in its early stage 51, 93. Here, we summarize the current circRNA-related studies in PC and list the deregulated circRNAs in **Table 2 and Figure 3**.

### Profiles of circRNA expression in PC

Extensive reports have confirmed that many circRNAs are deregulated in pancreatic ductal adenocarcinoma (PDAC). By analyzing the data from the GEO (No: GSE79634) database, 256 differentially expressed circRNAs (DECs) were identified, among which 115 and 141 were upregulated and downregulated, respectively, in PDAC tissues in comparison with matched normal tissues 94. Utilizing Arraystar Human CircRNA Array Analysis, Guo et al. identified 289 DECs between 20 PC tissues and corresponding paracancerous tissues, of which 128 were upregulated and 161 were downregulated 95. Subsequently, the qRT-PCR results were consistent with the microarray data 95. A recent study identified more than 40,000 unknown circRNAs that have not been previously described in the circBase database via RNA sequencing analyses 96. More importantly, the authors also discovered a novel circRNA, “circPDAC”, that was produced from the 5' end of exon 3 of the noncoding RNA LOC107987178 and the 3' end of exon 3 of the adjacent noncoding RNA LOC100507377 96. This circRNA was significantly overexpressed in PC tissues and cells, whereas it was barely detected in normal pancreas tissues 96. Similarly, Li and colleagues identified 351 DECs between six paired PDAC tissues and normal tissues by utilizing circRNA microarray analysis, of which 209 and 142 circRNAs were upregulated and downregulated, respectively, in tumor samples 97. Based on two GEO microarray datasets, Xiao et al identified 289 and 170 DECs in GSE79634 and GSE69362, respectively; analysis using publicly available circRNA and miRNA databases of the top ten DECs revealed that hsa_circ_0007767 and hsa_circ_0092367 play essential roles in PDAC by acting as miRNA sponges 98. Additionally, Wong et al. performed circRNA sequencing to identify DECs between PANC-1 and SW1990 PC cells and nontumor human pancreatic ductal epithelial (HPDE) cells. Overall, 17,158 circRNAs were identified, of which 84% were EcircRNAs (GEO; No: GSE135731). Among these circRNAs, 83 upregulated and 86 downregulated circRNAs were found in PANC-1 and SW1990 cells compared with HPDE cells 71.

However, only a very small proportion of the DECs in these expression profiles have been verified to regulate carcinogenesis in PDAC. Although numerous efforts have been made to discover more circRNAs in PDAC, the study of PDAC-related circRNAs still faces great challenges.

### CircRNAs alter the proliferation and progression of PC

Numerous reports have highlighted that circRNAs alter proliferation, migration, invasion, metastasis, apoptosis, and the cell cycle by acting as oncogenes or tumor suppressors in PC. Multiple mechanisms, including miRNA sponge activity, cancer-related signaling pathway regulation, and interaction with proteins, are related to these functions. Therefore, miRNA sponges are still the leading mechanism related to circRNAs in PC.

### Oncogene

A previous study showed upregulated ciRS-7 in PC tissues, which was positively associated with LNM and venous invasion. In general, ciRS-7 enhanced the proliferation and invasion of PC by sponging miR-7 and inhibiting its activity, subsequently activating the EGFR and STAT3 signaling pathways 18. CircFOXK2 was markedly overexpressed in both PDAC cells and tissues. Silencing circFOXK2 significantly inhibited migration, invasion, liver metastasis and tumor growth in PDAC 71. CircFOXK2 competitively sponges miR-942 to increase the expression of its target genes ANK1, GDNF, and PAX6 71, 99. Notably, circFOXK2 interacted with YBX1 and hnRNPK to enhance the expression of the oncogenic proteins NUF2 and PDXK in PDAC 71. Moreover, the increase in NUF2 and PDXK expression caused by the overexpression of circFOXK2 was attenuated by knockdown of YBX1 and hnRNPK 71.

Another novel study found that overexpression of circ_0007534 promoted the proliferation, migration, and invasion while inhibiting the apoptosis of PDAC cells by sponging miR‐625 and miR‐892b 19, 100. Circ_100782 was overexpressed in PC tissues. Circ_100782 knockdown effectively inhibited proliferation through the IL6-STAT3 pathway by directly sponging miR-124 in PC cells 101. Circ-BFAR is overexpressed in PDAC patients 65, and its knockdown significantly inhibits the proliferation, migration, invasion, tumor growth and metastasis of PDAC cells 65. Circ-BFAR enhanced the expression of its target gene mesenchymal-epithelial transition factor (MET) by absorbing miR-34b-5p in PC cells. MET is frequently overexpressed and acts as an oncoprotein in PC 102. Collectively, these findings highlighted the significance of the circ-BFAR/miR-34b-5p/MET axis in the progression of PDAC and demonstrated that circ-BFAR performs an oncogenic function and might function as a possible diagnostic biomarker and therapeutic target in PDAC 65.

Circ-ASH2L was overexpressed in PDAC tissues and cells 103. Based on bioinformatic analysis and experiments, circ-ASH2L was confirmed to promote invasion, proliferation and angiogenesis by sponging miR-34a to enhance the level of Notch 1 103. A recent study found that hsa_circ_001653 was upregulated in PDAC tissues and cells. The knockdown of hsa_circ_001653 inhibited miR-377-targeted HOXC6 and suppressed PDAC cell proliferation, invasion, angiogenesis, and tumorigenesis whereas promoting cell apoptosis 104. The upregulation of human HOXC6 in PCa is related to tumor progression and serves as an independent prognostic biomarker 105. Zhu et al. revealed that overexpression of hsa_circ_0006215 enhanced SERPINA4 expression by absorbing miR-378a-3p, therefore initiating and promoting the occurrence and progression of PC 106. Hsa_circ_0005397 (termed as circRHOT1) knockdown inhibited the cell proliferation, migration and invasion of PDAC cells. Bioinformatic analysis revealed that circRHOT1 can function as a miRNA sponge for miR-26b, miR-125a, miR-330 and miR-382 to affect various cancer-related pathways in PDAC 107. Interestingly, another study confirmed that circRHOT1 promoted proliferation, apoptosis and invasion by downregulating the expression of miR-125a-3p to increase the expression of its target gene E2F3 in PC 108. Zhang et al found that the expression of circ_0075829 was significantly overexpressed in PC tissues compared with adjacent noncancerous tissues and was associated with tumor size and lymphatic metastasis 109. Circ_0075829 significantly promoted the proliferation and metastasis of PC cells by directly sponging miR-1287-5p and elevating the expression of LAMTOR3 109.

Microarray analysis and qRT-PCR results confirmed that circZMYM2 (hsa_circ_0099999) was markedly upregulated in human PC tissues and cells 110. The overexpression of circZMYM2 promoted PC cell proliferation and invasion and inhibited apoptosis, while the knockdown of circZMYM2 had opposite effects. Moreover, circZMYM2 knockdown attenuated PC cell tumor formation and growth *in vivo* 110. Importantly, circZMYM2 downregulates the expression of miR-335-5p, which is a crucial factor that suppresses PC progression by inhibiting JMJD2C 110. MJD2/KDM4, which is a member of the JMJ family, is involved in the proliferation and progression of PC and transforms abnormal cells into invasive and metastatic forms by enhancing cell invasive and migratory abilities 111. Briefly, circZMYM2 knockdown can increase the expression of miR-335-5p to subsequently attenuate JMJD2C and inhibit the progression of PC 110. Xing et al. detected low levels of miR-217 and high levels of circ-ADAM9 in PC tissues and cells 112. Of note, the knockdown of circ-ADAM9 dramatically suppressed the cell growth, migration, and invasion of PC *in vitro* and inhibited tumor growth *in vivo* 112. Mechanistically, circ-ADAM9 directly sponges miR-217 to suppress its effect on its target serine protease 3 (PRSS3) and then indirectly activates the ERK/VEGF pathway. PRSS3 is an oncogene that is significantly overexpressed in PC 112. It was suggested that circ-ADAM9 may be an oncogene influencing cancer growth and progression through the miR-217/PRSS3 axis 112. Xu et al. discovered that elevated circSFMBT1 (hsa_circ_0066147) enhanced the proliferation, invasion, migration, and EMT process of PC cells and inhibited the apoptosis of PC cells *in vitro* by regulating the miR-330-5p/PAK1 pathway by sponging miR-330-5p 113. Moreover, circSFMBT1 knockdown suppressed the growth of tumor and lung metastases *in vivo* through the miR-330-5p/PAK1 axis 113.

### Tumor suppressors

Hsa_circ_0086375, generated from the NFIB1 gene (circNFIB1), was obviously downregulated in PC tissues in comparation with adjacent normal tissues and negatively correlated with LNM in PC patients 66. CircNFIB1 depletion increased the expression and secretion of VEGF-C in PC cells 66. VEGF-C is a VEGFR3 ligand that participate in lymphangiogenesis and is considered the upstream regulator of the PI3K/Akt signaling pathway 114, 115. Furthermore, *in vitro* experiments showed that conditioned medium from circNFIB1-knockdown PC cells dramatically enhanced HLEC tube formation and migration, whereas conditioned medium from circNFIB1-overexpressing PC cells exerted the opposite effects 66. *In vivo* experiments showed that circNFIB1 knockdown markedly enhanced LNM in PC cells 66. Importantly, circ-NFIB1 directly absorbs miR-486-5p to attenuate the oncogenic function of miR-486-5p to some degree and subsequently upregulates the expression of the miR-486-5p target PI3K p85α, a regulatory subunit of PI3K (PIK3R1) 66. Circ-NFIB1-induced VEGF-C attenuated the activation of the PI3K/Akt signaling pathway and suppressed the lymphangiogenesis and LN metastasis of PC 66. Hsa_circ_001587 expression was markedly lower in PDAC cells and tissues. Hsa_circRNA_001587 overexpression inhibited proliferation, angiogenesis, tumorigenesis, migration and invasion abilities by decreasing the expression of MMP-2, MMP-9, MCM2 and VEGF in PC 116. Mechanistic studies suggested that hsa_circRNA_001587 directly sponges miR-223 to enhance the level of its target gene SLC4A4, a cancer-promoting gene 116. Jiang et al discovered that hsa_circ_0001649 is also aberrantly downregulated in both PDAC tissues and cells 117. Moreover, hsa_circ_0001649 overexpression obviously suppressed the proliferation and colony formation abilities and enhanced the apoptosis rate of PDAC cells 117.

### CircRNAs function as diagnostic and prognostic biomarkers in pancreatic cancer

Most PC patients present with symptomatic, surgically unresectable disease due to a lack of dependable and valid early diagnostic techniques. Therefore, achieving early detection of PC is important and would result in a significant improvement in overall survival (OS) 4. Unlike their linear counterparts, circRNAs have a unique stable closed loop structure, which contributes to their stable expression in tissues, saliva, plasma, and exosomes 118. Additionally, circRNAs may function as special molecular markers in cancers because of their abundance, conservation and specificity in tissues and cells 25, 119, 120.

To date, several investigations have confirmed the roles of circRNAs in different cancers, including PC 107, 121, 122, 123. As mentioned above, it was found that high expression of circ_0007534 was correlated with poor prognosis in PC patients 104. Patients with lower expression of circ-ADAM9 had a better OS rate than those with higher expression of circ-ADAM9 (*p=*0.001) 112. High expression of circ-ASH2L was positively correlated with tumor malignancy, lymphatic invasion and TNM stage 103.

Yao's study revealed that PC patients whose tumors expressed high levels of circ-LDLRAD3 (hsa_circ_0006988) had a worse prognosis (*p=*0.0476) 124. Circ-LDLRAD3 overexpression could act as an oncogene by sponging miR-137-3p to promote the cell proliferation, migration and invasion of PC 124. Interestingly, another study also confirmed that circ-LDLRAD3 was significantly upregulated in PC tissues and plasma 122. A high level of circ-LDLRAD3 was positively associated with tumor venous invasion (*p=*0.025) and lymphatic metastasis (*p=*0.014) 122, and its expression in plasma was significantly related to CA19-9 levels (*p=*0.03), N stage (*p=*0.049), venous invasion (*p=*0.005), and lymphatic metastasis (*p=*0.014) in PC tissues 122. Additionally, circ-LDLRAD3 coupled with CA19-9 was confirmed to have higher sensitivity and specificity for the diagnosis of PC 122. Therefore, these findings suggest that circ-LDLRAD3 may function as a novel biomarker for the diagnosis of PC 122. A high-throughput circRNA microarray showed that circ_0030235 is highly expressed in PDAC tissue samples 97. qRT-PCR further confirmed that circ_0030235 was also markedly elevated in PDAC tissues and cells compared to paired nontumorous tissue specimens and HPDE cells, respectively 121. In PDAC tissues, high expression of circ_0030235 was confirmed as a possible biomarker for poor prognosis by Kaplan-Meier (KM) analysis (*p=*0.001) 121. Moreover, high expression of circ_0030235 is an independent prognostic indicator of unfavorable OS for PDAC patients according to a multivariate Cox analysis 121.

Conversely, as mentioned above, PDAC patients with a high level of hsa_circ_0001649 had a higher OS rate (*p=*0.002) 117. It was also found that PDAC patients with low levels of hsa_circ_0001649 presented with more advanced tumor stage (*p=*0.038) and lower differentiation grade (*p=*0.018) 117. The univariate analysis of OS verified that high hsa_circ_0001649 expression (*p=*0.003) and high differentiation grade (*p=*0.006) were all good prognostic indicators 117. Furthermore, the Cox proportional hazards model demonstrated that hsa_circ_0001649 may serve as an independent prognostic predictor of OS in PDAC patients (*p=*0.039) 117.

Exosomes are a type of nanosized (30-150 nm) extracellular vesicle with a lipid bilayer membrane released by multiple cell types and can be detected in various bodily fluids, such as plasma, saliva, and urine 125, 126. As crucial mediators of intercellular communication, exosomes participate in carcinogenesis and cancer progression 125, 127. Recent studies have identified the abundance and stability of circRNAs in exosomes **(Figure 2G)** 128. Hence, exosomal circRNAs might be potential biomarkers for the detection of some cancers 128. For instance, circ-IARS located within exosomes, generated and released by PC cells, was obviously overexpressed in the plasma exosomes of patients with metastasis and in PC patient tissue 129. A high level of circ-IARS was positively associated with liver metastasis (*p=*0.000), vascular invasion (*p=*0.038), and TNM stage (*p=*0.011) 129. PC patients with higher circ-IARS expression showed a lower OS rate than those with lower circ-IARS expression (*p=*0.01) 129. Furthermore, it was found that circ-IARS enters HUVECs via exosomes to enhance cancer metastasis 129. Collectively, these findings show that circ-IARS carried by exosomes from PC cells was taken up by HUVECs, specifically sponging miR-122 in HUVECs to relieve its inhibition of the target gene RhoA 129. This further elevated the expression and activity of RhoA, thereby reducing the expression of the tight junction protein ZO-1 and increasing the expression of F-actin and endothelial monolayer permeability 129. Another study identified the tumor-released exosomal circ-PDE8A (hsa_circ_0036627) in the exosomes of PC cells from patients with liver metastasis by using human circRNA microarrays 130. Circ-PDE8A levels were significantly higher in PC tissues than in adjacent normal tissues. It was also discovered that the level of circ-PDE8A was related to lymphatic invasion (*p=*0.014), T factor (*p=*0.049) and TNM stage (*p=*0.005) in PC patients 130. PC patients with higher levels of circ-PDE8A had a significantly poorer OS than those with lower expression of circ-PDE8A (*p=*0.016) 130. Further investigations verified that circ-PDE8A absorbed miR-338 to enhance the growth and invasion of PC cells by upregulating the expression level of MET 130, 131. In addition, KM survival curves demonstrated that the high expression of circ-PDE8A in plasma exosomes predicted worse OS in PC patients (*p=*0.011) 130. Based on these findings, exosomal circRNAs may function as potential biomarkers for the diagnosis, prognostic prediction and progression of PC 129, 130.

### CircRNAs as therapeutic targets in pancreatic cancer

Recent studies have determined that circRNAs affect the sensitivity of PC to chemotherapy 13, 132, 133. Gemcitabine (GEM) is currently an effective monotherapy for the treatment of advanced PC. Nevertheless, acquired GEM resistance has resulted in treatment failures in a large number of PC patients 134. Xu et al. detected 12,866 circRNAs and identified 81 DECs between GEM-resistant SW1990 cells and parental SW1990 cells by using circRNA microarrays 135. Of the 81 DECs, 26 were upregulated and 55 were downregulated 135. Moreover, circRNA_101672 and circRNA_004077 were the top two upregulated circRNAs and were both located on chromosome 16, which may make a difference in GEM resistance in PC. RNA sequencing analysis demonstrated that 68 and 58 circRNAs were upregulated and downregulated, respectively, in PANC-1-GR cells in comparation with control PANC-1 cells 13. qRT-PCR experiments demonstrated that although the expression trends of the top 10 DECs were consistent with the microarray data, only two of them (chr14:101402109-101464448C and chr4:52729603-52780244C) were verified to be the most markedly upregulated in PANC-1-GR cells 13. Similarly, it was confirmed that these two most significantly upregulated circRNAs were also upregulated in plasma samples of GEM-nonresponsive PDAC patients but not in GEM-responsive PDAC patients 13. More importantly, knockdown and overexpression of these two circRNAs enhanced the GEM sensitivity of PANC-1-GR cells and GEM resistance of PANC-1 and MIA PACA-2 cells 13. In addition, GO and pathway analyses of the parental genes related to the DECs revealed eight significantly enriched pathways, among which the VEGF and ErbB signaling pathways were previously confirmed to participate in GEM resistance 136 and PDAC progression 137. All these findings suggest that in addition to the two most significantly upregulated circRNAs that have been identified, more circRNAs might take part in the GEM resistance of PANC-1-GR cells 13. CircHIPK3 (hsa_circ_0000284) was proposed to be involved in tumorigenesis and chemotherapy resistance in different cancers 138, 139, 140, 141. In a recent study, Zhu's team discovered that circHIPK3 was elevated in PC tissues and cells with GEM resistance 132. Briefly, circHIPK3 absorbed miR-330-5p to enhance the resistance of GEM and regulate proliferation and progression by upregulating the expression of RASSF1 in PC 132.

## Conclusions and Prospects

Due to late diagnosis and low response to chemotherapy, PC patients have poor prognosis, and the specific pathogenesis of PC is still unclear. Hence, it is of paramount importance to identify earlier diagnostic and more efficient therapeutic approaches for the clinical management of PC. Initially, considered to be RNA splicing errors, circRNAs have generated increasing attention due to their strong correlation with different physiological and pathological processes, their important roles in various diseases, and their high degree of tissue and development specificity 142. Currently, many studies have revealed the value of circRNAs in clinical practice in various tumors, including PC. In this review, circRNAs were shown to participate in different biological processes of PC and serve as promising biomarkers for the diagnosis, prognosis, response to chemotherapy, and risk evaluation of PC. At present, m6A-modified circRNAs can promote the transport of circRNAs to cytoplasm 143, protein translation 81, and degradation processes 144. However, there has not been a study involving m6A modification of circRNA in PC, so it is worth exploring in later circRNA research on PC. In fact, research on the field of circRNAs in PC is still in its early stage compared with miRNA and long noncoding RNA (lncRNA) research, and only a tiny proportion of important circRNAs in PC have been identified and characterized. Herein, some suggestions are put forward for future research on circRNAs in PC. First, although many scholars have initially proposed models related to circRNA formation, the detailed mechanism of circRNA formation is far from understood. More scientific explorations and efforts are imperatively needed to fully illustrate the mechanisms of biogenesis, turnover and degradation of circRNAs. A thorough annotation of circRNA biogenesis and regulation will undoubtedly strengthen our understanding of circRNA functions. Second, in PC, circRNAs exert their effects mainly by serving as miRNA sponges. Nonetheless, because a large number of circRNAs have much lower abundance than miRNAs and there are only a few circRNAs with many MREs 145 , the miRNA sponge mechanism for circRNA is faced with a great dilemma. Therefore, it is imperative to further elucidate other mechanisms by which circRNAs function, including gene transcription regulation, interaction with RBPs and translation potential. Third, the detection of circRNAs is currently mainly performed in clinical tissue specimens. In future research, the expression of circRNAs should be detected in more extensive clinical samples related to the disease, such as blood, cerebrospinal fluid, urine, and saliva. Studies can also utilize combined detection methods to obtain better diagnostic value, including combining the detection methods for various circRNAs with traditional diagnostic markers related to tumors. Nevertheless, whether circRNAs can successfully function as effective biomarkers for the diagnosis and prognosis of cancers is still far from clinical application. Fourth, future studies may regard circRNAs as potential targets for the treatment of tumors. How to deliver circRNAs to the corresponding parts of the body, how to ensure longstanding and effective function, and how to avoid the occurrence of immune rejection are all difficult problems that need to be resolved. Last, the application of specific circRNAs related to human diseases in the treatment of tumors is the ultimate goal of circRNA-related research. Therefore, more controlled clinical studies and experiments need to be conducted on a large scale in tumor patients.

In summary, the current understanding of circRNA functions in PC is still very limited. Fortunately, following the accelerated advancement of high-throughput RNA sequencing and biotechnology 42 and the numerous available online databases, more circRNAs will be identified and validated. In the near future, we believe that an in-depth understanding of the characteristics of circRNAs and correct application of circRNAs in clinical practice will represent a giant leap in the treatment of PC.

## Figures and Tables

**Table 1:** Online databases related to circRNAs

Database	Website	Description	References
CircBase	http://www.circbase.org/	A comprehensive database that provides published circRNAs in different species (human, mouse, *C. elegans*, and Latimeria organisms) and identification of circRNAs.	46
Circ2Traits	http://gyanxet-beta.com/circdb/	A comprehensive knowledgebase of the potential association of circRNAs with diseases and traits in humans.	47
CIRCpedia v2	http://www.picb.ac.cn/rnomics/circpedia	A database for comprehensive circRNA annotations and expression comparisons from over 180 RNA-seq datasets across six different species.	48
CircInteractome	http://circinteractome.nia.nih.gov	A database to explore the possible interactions of circRNAs with miRNAs and RBPs.	49
CircNet	http://circnet.mbc.nctu.edu.tw	A useful tool to investigate the regulatory relationship between circRNAs, miRNAs and genes.	50
CircRNADb	http://reprod.njmu.edu.cn/circrnadb	A database to provide detailed information on circRNAs including genome sequence, exon splicing, ORF, IRES, and references to predict the translation potential of certain circRNAs.	44
TransCirc	https://www.biosino.org/transcirc/	A database used to predict the potential of all circRNAs to encode functional peptides.	45
MiOncoCirc	mioncocirc.github.io	A database compiled from clinical cancer samples that provides the expression of a certain circRNA in different cancer clinical samples.	51
TSCD	http://gb.whu.edu.cn/TSCD	A database used to characterize the features of human and mouse tissue-specific circRNAs.	52
CSCD	http://gb.whu.edu.cn/CSCD	A cancer-specific circRNA database contributes to the study of the function and regulation of cancer-related circRNAs.	53
exoRBase	http://www.exoRBase.org	A database containing more than 58000 circRNAs from 87 human blood exosomal RNA-seq datasets that provides circRNA annotation and expression levels and can assist researchers to discover new exosomal biomarkers for human diseases.	54
CircR2Disease	http://cgga.org.cn:9091/circRNADisease/	CircR2Disease is a comprehensive database for circRNAs dysregulated in different diseases and contains indicators showing that circRNAs participate in gene posttranscriptional regulation.	55

**Table 2:** Deregulated circRNAs in pancreatic cancer

CircRNAs	Expression change	Roles in PC	Putative function	Possible mechanism	Relationships with clinical features	Clinical association	Reference
circPDAC	Up	Unknown	Unknown	Unknown	LNM, TNM stage	A noninvasive biomarker	96
ciRS-7	Up	Oncogene	Promotes proliferation, invasion, metastasis	miRNA sponge (ciRS-7/miR-7 EGFR/STAT3 pathway)	LNM, tumor venous invasion	Unknown	18
circFOXK2	Up	Oncogene	Promotes growth, migration, invasion, liver metastasis	miRNA sponge (circFOXK2/miR-942/(ANK1, GDNF, PAX6); interaction with YBX1 and hnRNPK	Unknown	Unknown	71
hsa_circ_0007534	Up	Oncogene	Promotes proliferation, migration, invasion. Inhibits apoptosis.	miRNA sponges (circ_000753/miR-625 and miR-892b	Tumor stage, lymphatic invasion	OS	100
hsa_circ_100782	Up	Oncogene	Promotes proliferation	miRNA sponge (hsa_circ_100782/miR-124/IL6-STAT3 pathway)	Unknown	Unknown	101
circ-BFAR	Up	Oncogene	Promotes proliferation, migration, invasion, metastasis	miRNA sponges (circ-BFAR/miR-34b-5p/MET/Akt axis)	TNM stage	OS DFS	65
circ-ASH2L	Up	Oncogene	Promotes invasion, proliferation, angiogenesis	miRNA sponge (circ-ASH2L/miR-34a/Notch1 axis)	Lymphatic invasion, TNM stage	OS	103
hsa_circ_001653	Up	Oncogene	Promotes proliferation, invasion, tumorigenesis angiogenesis. Inhibits apoptosis.	miRNA sponge (hsa_circ_001653/miR-377/HOXC6 axis)	Unknown	OS	104
hsa_circ_0006215	Up	Oncogene	Promotes growth, migration. Inhibits apoptosis.	miRNA sponge (hsa_circ_0006215/miR-378a 3p/SERPINA4 axis)	Unknown	Unknown	106
circ-ADAM9	Up	Oncogene	Promotes proliferation, migration and invasion	miRNA sponge (circADAM9/miR-217/PRSS3 axis)	Lymphatic metastasis, TNM stage	OS	112
circRHOT1	Up	Oncogene	Promotes proliferation, invasion, migration	miRNA sponge (circRHOT1/miR-26b, miR-125a, miR-330 and miR-382)	Unknown	Unknown	107
circRHOT1	Up	Oncogene	Promotes proliferation, migration, invasion. Inhibits apoptosis	miRNA sponge (circRHOT1/miR-125a-3p/E2F3 axis)	Lymphatic metastasis	Unknown	108
circ_0075829	Up	Oncogene	Promotes proliferation, migration and invasion, tumorigenicity and metastasis	miRNA sponge (circ_0075829/miR-1287-5p/LAMTOR3 axis)	Tumor size, lymphatic metastasis	Unknown	109
circZMYM2	Up	Oncogene	Promotes proliferation and invasion. Inhibits apoptosis	miRNA sponge (circZMYM2/miR-335-5p/JMJD2C axis)	LNM	Unknown	110
circSFMBT1	Up	Oncogene	Promotes proliferation, migration, invasion, EMT, metastasis. Inhibits apoptosis.	miRNA sponge (circSFMBT1/miR-330-5p/PAK1 axis)	Unknown	Unknown	113
circNFIB1	Down	Tumor Suppressor	Inhibits lymphangiogenesis, LNM, tumorigenesis	miRNA sponge (circNFIB1/miR-486-5p/PIK3R1/VEGF-C axis)	LNM	OS DFS	66
hsa_circ_001587	Down	Tumor Suppressor	Inhibits proliferation, migration, invasion, angiogenesis, tumorigenesis	miRNA sponge (hsa_circ_001587/miR-22/SLC4A4 axis)	Unknown	Unknown	116
hsa_circ_0001649	Down	Tumor Suppressor, Biomarker	Inhibits proliferation, colony-forming ability. Promotes apoptosis.	Unknown	TNM stage, differentiation grade	OS	117
circ-LDLRAD3	Up	Oncogene	Promotes proliferation, migration, invasion	miRNA sponge (circ-LDLRAD3/miR-137-3p/PTN axis)	Unknown	OS	124
circ-LDLRAD3	Up	Biomarker	Unknown	Unknown	Tissue samples (venous invasion, lymphatic invasion); plasma samples (CA19-9 level, N stage, venous invasion, lymphatic metastasis)	Combination with CA19-9 increased the diagnostic value in PC	122
hsa_circ_0030235	Up	Oncogene	Promotes growth, migration, invasion. Inhibits apoptosis.	miRNA sponge (circ_0030235/miR-1253 and miR-1294)	Lymphatic invasion, TNM stage	OS	121
circ-IARS	Up	Oncogene	Promotes metastasis	miRNA sponge (circ-IARS/miR-122/RhoA/F-actin and ZO-1 axis)	Differentiation grade, vascular invasion, liver metastasis, TNM stage	OS; circ-IARS in exosomes as a marker for the early diagnosis and prognostic prediction in PC	129
circ-PDE8A	Up	Oncogene, Biomarker	Promotes invasion, growth, liver metastasis	miRNA sponge (circ-PDE8A/miR-338/MACC1/MET axis)	Lymphatic invasion, T factor, TNM stage	OS (both in PC patient tissues and plasma exosomes)	130
circRNA_101672, circRNA_004077	Up	Unknown	Enhances GEM resistance	Unknown	Unknown	Unknown	135
chr14:101402109-101464448C, chr4:52729603-52780244C	Up	Unknown	Enhances GEM resistance	Possibly related to the ErbB and VEGF pathways	Unknown	Unknown	13
circHIPK3	Up	Oncogene, Biomarker	Promotes proliferation, invasion, migration, EMT. Inhibits apoptosis. Enhances GEM resistance	miRNA sponge (circHIPK3/miR-330-5p/RASSF1 axis)	Unknown	OS	132

## Abbreviations

ceRNA: competing endogenous RNA
miR: miRNA
MREs: miRNA response elements
EGFR: epidermal growth factor receptor
HuR: human antigen R
Amotl1: angiomotin-like1
YBX1: Y-box binding protein
hnRNPK: heterogeneous nuclear ribonucleoprotein K
PDXK: pyridoxal kinase
NUF2: NUF2 component of NDC80 kinetochore complex
ALKBH5: alkB homologue 5
FAAH: fatty acid amide hydrolase
CDK2: cyclin-dependent kinase 2
P21: cyclin-dependent kinase inhibitor 1
ANKRD52: ankyrin repeat domain 52
RNA Pol II: RNA polymerase II
U1 snRNP: U1 small nuclear ribonucleoprotein
PABPN1: poly (A)-binding protein nuclear 1
ORFs: open reading frames
IRES: internal ribosome entry site element
FTO: obesity-associated protein
METTL3/14: methyltransferase-like 3/14
GEO: Gene Expression Omnibus
LNM: lymph node metastasis
ANK1: ankyrin 1
LAMTOR3: lysosomal adaptor, MAPK and MTOR activator 3
GDNF: glial cell-derived neurotrophic factor
PAX6: paired box 6
HOXC6: homeobox C6
PCa: prostate cancer
SERPINA4: serpin family A member 4
PAK1: p21-activated kinase 1
VEGF-C: vascular endothelial growth factor C
VEGFR3: vascular endothelial growth factor receptor-3
HLEC: lymphatic endothelial cell
SLC4A4: solute carrier family 4 member 4
HUVECs: human microvascular vein endothelial cells
PANC-1-GR: GEM-resistant PANC-1
GO: Gene Ontology
RASSF1: ras association domain family member 1
